# ProtecT-2-D trial protocol: cardiovascular protection in patients with type 2 diabetes and established heart and/or vascular disease at a cardio-metabolic clinic—a randomized controlled trial

**DOI:** 10.1186/s12933-024-02340-w

**Published:** 2024-07-08

**Authors:** Katrine Schultz Overgaard, Roda Abdulkadir Mohamed, Thomas Rueskov Andersen, Jess Lambrechtsen, Kenneth Egstrup, Søren Auscher

**Affiliations:** 1 Cardiovascular Research Unit, Odense University Hospital Svendborg, Baagøes Allé 15, 5700 Svendborg, Denmark; 2 Department of Clinical Research, Faculty of Health Sciences, University of Southern Denmark, Campusvej 55, 5230 Odense M, Denmark

**Keywords:** Cardio-metabolic clinic, Type 2 diabetes, Cardiovascular disease, Heart disease, Vascular disease, Atherosclerosis, Coronary artery disease, Multidisciplinary, Decision-making algorithm

## Abstract

**Background:**

Cardiovascular disease remains the primary cause of morbidity and mortality despite advancements in the treatment of patients with type 2 diabetes. Effective diabetes management extends beyond blood glucose control and includes cardiovascular prevention and treatment. However, the conventional healthcare model often emphasizes single-disease-specific management, leading to fragmented care. We aim to establish an affordable Cardio-Metabolic Clinic (CMC) that can provide comprehensive assessment and specialized care with a focus on cardiovascular protection.

**Methods:**

The ProtecT-2-D study is a prospective, randomized control trial at the Cardiovascular Research Unit, Odense University Hospital Svendborg, Denmark. In this study, 1500 participants with type 2 diabetes and cardiovascular disease will be randomly assigned in a 2:1 ratio to receive either the intervention: treatment in the CMC, or the control: standard of care. The Cardio-Metabolic Clinic applies a decision-making algorithm coded with the latest guidelines to evaluate lifestyle factors and manage medical treatment. Health examinations are conducted at baseline and after three years, and clinical events will be assessed through registry and journal audits after five and ten years. The primary outcome is the time to the first occurrence of a composite of cardiovascular deaths, non-fatal acute myocardial infarctions, non-fatal stroke, or hospitalization due to heart failure at a time frame of five years.

**Discussion:**

The Cardio-Metabolic Clinic represents a pioneering approach to diabetes management that aims to improve patient outcomes by reducing the cardiovascular disease burden. This study could transform diabetes care and offer a multidisciplinary, cost-effective, and specialized treatment. We need to establish the efficacy and feasibility of a CMC to integrate comparable clinics into broader healthcare systems, and potentially enhance cardiovascular health in patients with type 2 diabetes.

**Trial registration number:**

ClinicalTrials.gov NCT06203860.

**Graphical Abstract:**

**Figure:**
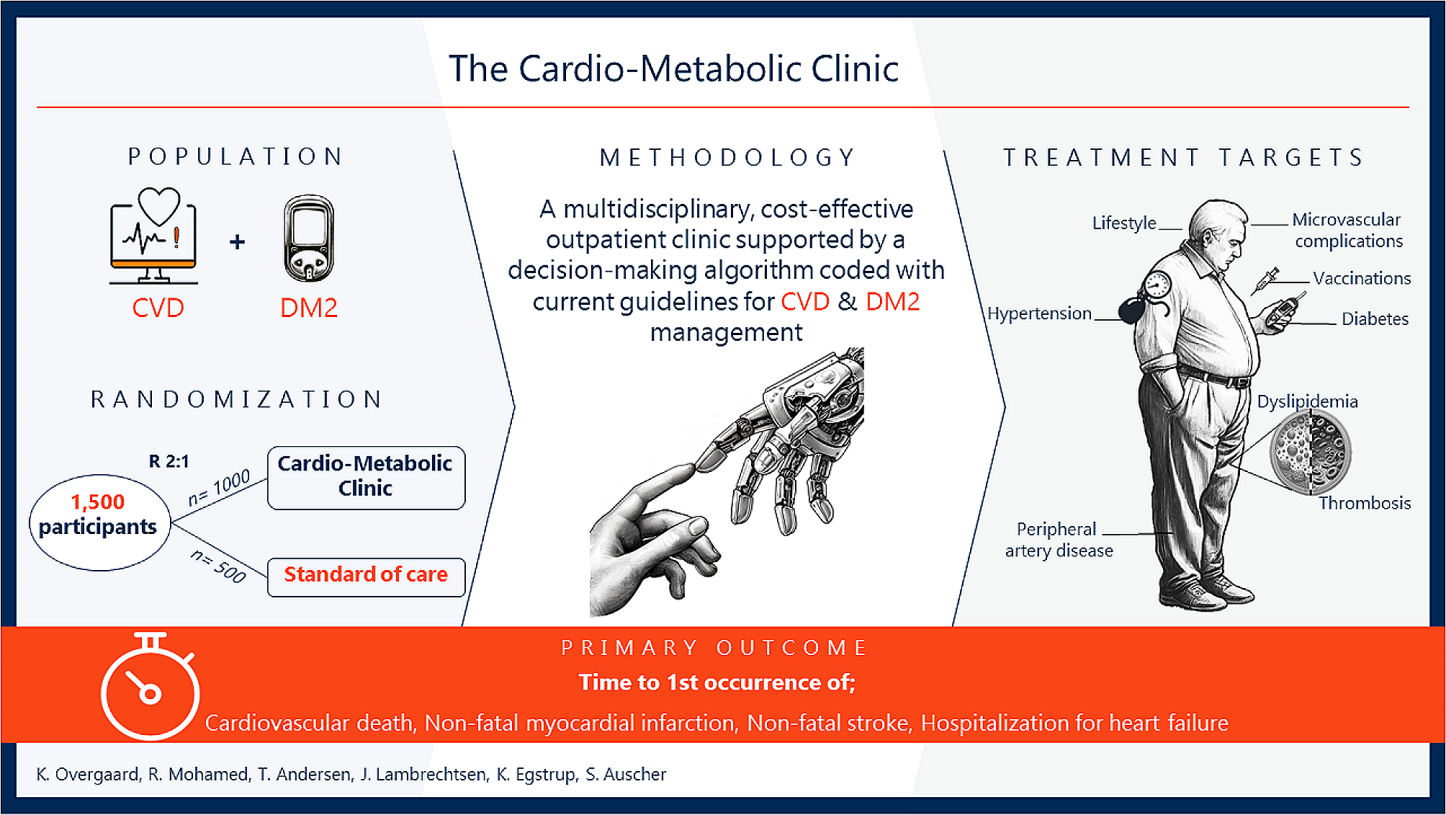

**Supplementary Information:**

The online version contains supplementary material available at 10.1186/s12933-024-02340-w.

## Background

The number of patients with type 2 diabetes (T2D) has more than tripled in the last twenty-five years, and every 20th person in the Danish population is currently living with T2D [1].

The risk of developing cardiovascular (CV) disease or premature death is 2–4 times higher in patients with T2D compared with the general population [2]. T2D is a systemic disease that is associated with both macro- and microvascular complications, and it is often accompanied by other metabolic disorders like hypertension, dyslipidemia, obesity, and metabolic dysfunction-associated steatohepatitis. A recent American study showed that half of T2D patients suffered from at least three other cardio-renal-metabolic conditions [3]. Patients with T2D also have an increased risk of developing heart failure and cardiomyopathy due to diabetes [4].

The STENO-2 study [5] demonstrated that multifactorial intervention on CV risk factors in patients with T2D significantly reduced mortality. Treatment with statins, angiotensin-converting-enzyme (ACE) inhibitors, and anti-hyperglycemic drugs are the cornerstone in the management of patients with T2D [6–8]. Similarly, the prospective RAMP study with more than 50,000 participants demonstrated a substantial reduction in CV risk (56.6%), microvascular complications (11.9%), and mortality (66.1%) through multifactorial diabetes and risk factor intervention compared to the standard of care [9].

New treatments for patient with diabetes have revolutionized the management of CV risk in patients with T2D and CV disease. Sodium-glucose co-transporter 2 inhibitors (SGLT2i) and glucagon-like peptide 1 receptor analogs (GLP1-RA) have been a “game changer” in diabetes management because they demonstrate significant reductions in CV morbidity and mortality in patients with established atherosclerotic CV disease, heart failure, and reduced kidney function [10–18]. Nevertheless, patients with T2D continue to face an increased risk of heart failure and CV death [2]. Patient adherence and physician management are critical in diabetes care. The EUROASPIRE IV and V studies [19, 20] revealed issues such as incomplete dose titration, slow treatment updates, and poor follow-up by physicians, contributing to suboptimal care. This is particularly notable among female patients who show higher rates of guideline non-adherence [21]. Furthermore, the healthcare system usually focuses on single-disease management. Patients with T2D are managed in specialty-focused outpatient clinics, which often results in fragmented care, inadequate treatment, higher costs, and worse CV outcomes [22, 23]. This emphasizes a need for a multidisciplinary and thorough assessment of patients with T2D and a closer collaboration between the specialties in internal medicine.

We propose a Cardio-Metabolic Clinic (CMC) that provides comprehensive assessment and specialized care following current guidelines. The concept of such clinics have previously been emphasized, which often involve collaboration between specialists in cardiology, endocrinology, and nephrology, which results in high costs [22, 24, 25]. A recent analysis questioned the cost-effectiveness of primary care based cardio-metabolic risk prevention programs [26]. Our goal is to develop an affordable and resource-efficient clinic model. Our CMC comprises of a decision-making algorithm to guide patient care and medical students or nurses that provide the daily patient contact under supervision of a cardiologist. Furthermore, we are broadening our clinical services to cover a wide range of cardiac conditions encountered in the outpatient cardiology clinic. This expansion includes patients with atrial fibrillation, severe hypertension, and valvular heart disease, along with the prevalent high-risk CV diseases.

## Methods

### Hypothesis

We hypothesize that a multidisciplinary approach in a Cardio-Metabolic Clinic will reduce the cardiovascular morbidity and mortality by 15% compared to the standard of care.

### Objectives

The primary objective of the ProtecT-2-D trial is to examine if a Cardio-Metabolic Clinic is superior to the standard of care in reducing cardiovascular morbidity and mortality in patients with T2D and established heart or vascular disease.

Secondary objectives are to evaluate the changes in microvascular and macrovascular complications, protocol-driven medication, patient experienced symptoms, and the cost-effectiveness of the CMC.

### Study setting

The ProtecT-2-D is a single-center, randomized, open label, prospective, controlled trial. The study is carried out at the Cardiovascular Research Unit at Odense University Hospital, Svendborg, Denmark.

### Study population

Eligible participants include male and female adults (> 18 years) with a confirmed diagnosis of T2D and established heart or vascular disease. This encompasses atherosclerotic disease defined as: prior acute coronary syndrome, chronic coronary syndrome, stroke, peripheral arterial disease, or imaging-verified ischemic heart disease. Additionally, patients with heart failure, atrial fibrillation/flutter, valvular heart disease, or hypertension treated with at least three antihypertensive drugs are eligible. Participants must understand all study procedures and provide informed consent. They cannot participate in other clinical trials involving investigational products or devices that might interfere with the study endpoints, have other types of diabetes, and must have a life expectancy of more than 5 years. A full list of patient eligibility criteria can be found in the study protocol (Supplementary material 1).

### Recruitment and screening

Recruitment will be performed by the dedicated project staff at the Cardiovascular Research Unit in Svendborg, Denmark. Potential participants are found according to the eligibility criteria and are identified from patients referred from general practices, or with an appointment in the outpatient clinic of Cardiology or Endocrinology at Svendborg Hospital. Participants are invited by electronic letter and can contact the study staff if they are interested in participating. Written material is sent to participants prior to the first visit. During the visit, participants receive oral information about the study, and informed consent is obtained before study-specific procedures begin. Participants are informed that they can decline participation or withdraw from the study at any time without consequences.

### Randomization

**Fig. 1:**
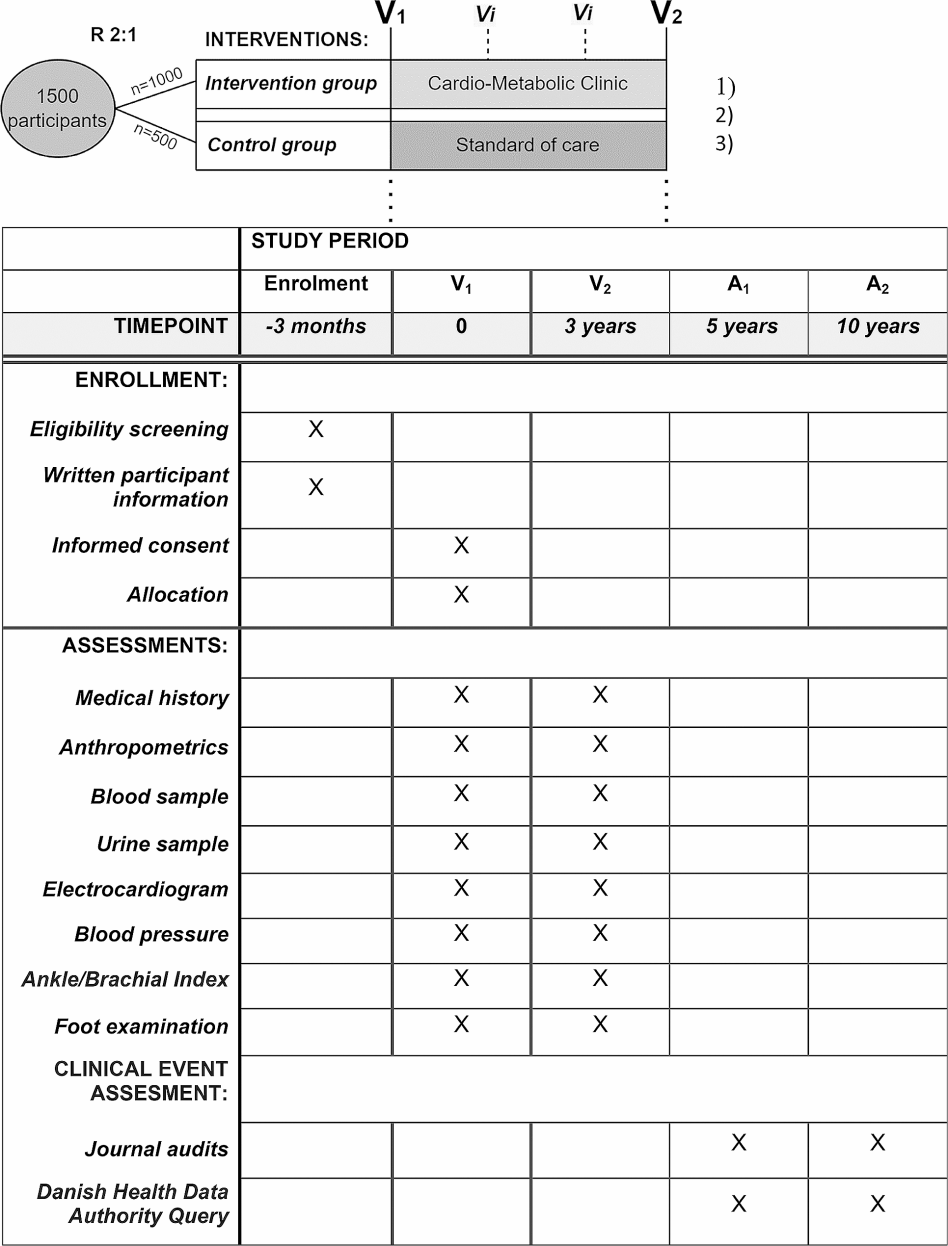
Trial design and time schedule. *V*_*1*_ Visit baseline, *V*_*2*_ Follow-up visit after 3-years, *V* Individual visits in the Cardio-Metabolic Clinic, *A*_*1*_ Clinical event assessment after 5 years post-intervention, *A*_*2*_ Clinical event assessment after 10 years post-intervention

Participants are randomized in a 2:1 ratio to one of the two arms, the intervention: Cardio-Metabolic Clinic (approximately 1000 patients), and the control: standard of care (approximately 500 patients) (*R* Fig. 1). Allocation is done at the first visit (*V*_*1*_ Fig. 1). Randomization is carried out using the Randomization Module in the electronic Case Report Form (e-CRF) system, REDCap. To ensure concealed allocation, the allocation table is created by a REDCap team member, who is independent of the ProtecT-2-D project staff.

### Baseline and follow-up

At visit 1 (*V*_*1*_ Fig. 1) participants will undergo a physical examination including medical history, anthropometrics, blood pressure, ankle/brachial index, foot examination and biochemical measurements. A detailed description of the examinations are provided in the protocol (Supplementary material 1). The baseline visit for participants in the control group is then completed, and they will be advised to continue diabetes management with their primary physician. Whereas participants in the intervention group, the CMC, follow a standardized evaluation and treatment program, further described in the next section. If a new treatment needs monitoring, individual visits are arranged (*V*_*i*_ Fig. 1). These can be telephone consultations or attendances. In some cases, additional biochemistry will be collected. All participants will be assessed at the three years follow-up visit and undergo the same examination program as the baseline visit (*V*_*2*_ Fig. 1). Assessments of clinical events will be conducted at an average of 5 and 10 years following the intervention (*A*_*1*_ + *A*_*2*_ Fig. 1). The occurrence of clinical events are assessed by journal audit and access to the Danish Health Data Authority.

### Intervention: the cardio-metabolic clinic

#### Organization

The Cardio-Metabolic Clinic consists of a low-cost model and is organized in three different layers centered on the patients. The innermost layer consists of medical students or specialized cardio-metabolic nurses, who maintains the daily patient contact and records the health examinations (*V*_*1*_*, V*_*i*_*,…, V*_*2*_ Fig. 1) to the e-CRF (REDcap). A decision-making algorithm activates upon randomization to the intervention arm with the purpose of offering tailored treatment recommendations. The second layer involves a review of patient risk profiles and the algorithm-recommended treatments by a cardiologist. The third layer is used in complex cases requiring multidisciplinary expertise, and it comprises an endocrinologist, nephrologist, and hepatologist.

#### Intervention

**Fig. 2:**
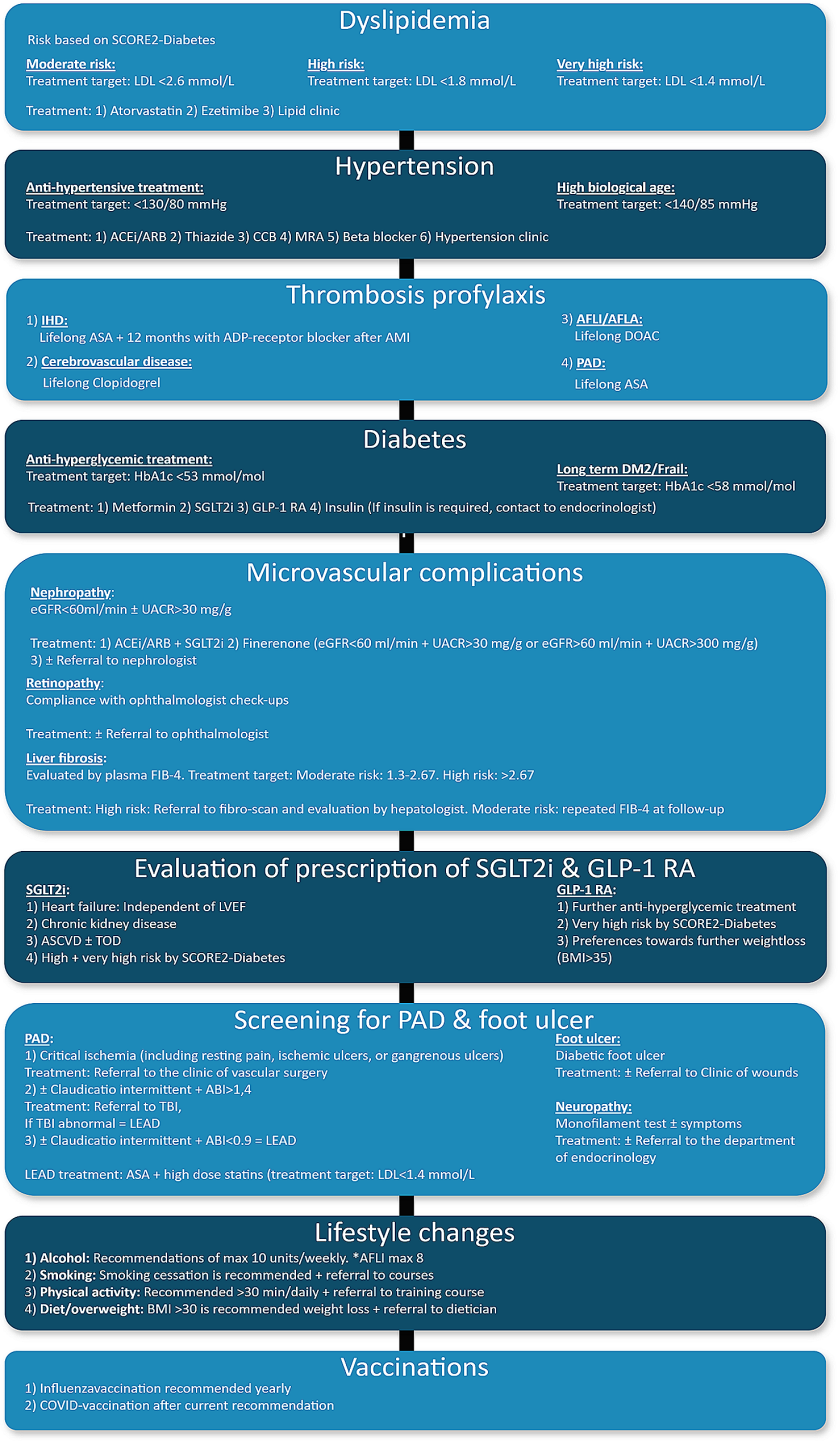
Overview of the treatment algorithm in the CMC. Participants in the intervention group, the CMC, will receive interventions targeting nine specific areas as shown in the figure. Participant’s risk of CVD is calculated using the SCORE2-Diabetes. The treatment algorithm is in accordance to the most recent guidelines from the European Society of Cardiology, therefore, there may be modifications over time. *LDL* Low-Density Lipoprotein, *ACEi* Angiotensin-Converting Enzyme inhibitor, *ARB* Angiotensin II Receptor Blocker, *CCB* Calcium Channel Blocker, *MRA* Mineralocorticoid Receptor Antagonist, *IHD* Ischemic Heart Disease, *ASA* Acetylsalicylic Acid, *ADP* Adenosine Diphosphate, *AFLI* Atrial Fibrillation, *AFLA* Atrial Flutter, *PAD* Peripheral Arterial Disease, HbA1c Hemoglobin A1c, SGLTi Sodium-Glucose Cotransporter Inhibitor, *GLP-1 RA* Glucagon-Like Peptide-1 Receptor Agonist, *DM* Diabetes Mellitus, *eGFR* Estimated Glomerular Filtration Rate, *UACR* Urinary Albumin-to-Creatinine Ratio, *FIB-4* Fibrosis-4 Score, *LVEF* Left Ventricular Ejection Fraction, *ASCVD* Atherosclerotic Cardiovascular Disease, *TOD* Target Organ Damage, *BMI* Body Mass Index, *ABI* Ankle-Brachial Index, *TBI* Toe-Brachial Index, *LEAD* Lower Extremity Arterial Disease, *COVID* Coronavirus Disease

Participants in the CMC are evaluated with a decision-making treatment algorithm in the REDcap-system. The treatment algorithm is coded in accordance to the most recent guidelines and recommendations for diabetes and CVD management [34]. Consequently, there may be modifications over time. Participants are categorized into cardiovascular risk groups using the SCORE2-Diabetes, a 10-year cardiovascular risk prediction model [34]. They receive interventions targeting nine specific areas (Fig. 2); dyslipidemia, hypertension, thrombosis prophylaxis, diabetes management, assessment of microvascular complications, evaluation of SGLT2-i and/or GLP1-RA, screening for peripheral artery disease, lifestyle modifications, and vaccinations. A detailed description of the treatment algorithm for the CMC can be found in the study protocol (Supplementary material 1). The interventions of CMC are considered complete when a participant is fully uptitrated on all targeted treatment areas.

### Outcomes

The effect of the CMC on the specified endpoints will be assessed as the between-group (CMC vs. standard of care) difference in change from baseline to follow-up at 3 years, and for the clinical events as journal audits and queries after 5 and 10 years. A detailed description of the study outcomes is provided in the study protocol (Supplementary material 1).

#### Primary outcome

The primary endpoint is the time to the first occurrence of a composite of CV death, non-fatal myocardial infarction, non-fatal stroke, and hospitalization for heart failure after 5 years.

#### Secondary outcomes

- The time to the first occurrence of the primary endpoint after 10 years.
- The time to the first occurrence of a one of the single components of the primary endpoint.
- A combination of the single components of the primary endpoint to assess the total symptom burden.
- Assessment of changes in micro- and macrovascular complications after 3 years.
- Assessment of protocol driven medical changes.
- Assessment of patient experienced symptoms.
- Cost-effectiveness analyses of the CMC.

### Timeline

The first participant was enrolled in January 2024, and we expect to enroll the last patient in January 2028. All participants will have a follow-up visit after three years where baseline assessments will be repeated. Clinical events will be assessed at 5- and 10-years follow-up by journal audits and access to the Danish Health Data Authority. Thus, the 5 years follow-up will be around January 2031 and 10 years follow-up in January 2036.

### Statistics

Sample size is based on the primary endpoint (the time to the first occurrence of cardiovascular mortality and morbidity). We anticipate a reduction in the primary endpoint of 15% in patients with T2D and established CVD assessed in the CMC compared to the standard of care. With a power of 80% and an alpha value of 0.05, 1306 participants are needed. We anticipate a dropout rate of 10–15%, and therefore 1500 participants will have to be included in the study.

The primary and secondary outcomes will be analyzed using cumulative incidence function for the composite primary outcome as well as each secondary outcomes. Furthermore, we will investigate CV-caused mortality through Kaplan Meier estimates. For the secondary numerical outcomes mixed-effects linear regression will be used. This will include a fixed effect for a time point, and a fixed effect for baseline values of the outcome. For each linear mixed effects model, a random intercept will be included for each enrolled patient. These outcomes will be measured at baseline and at three-year follow-up for the group. Secondary categorical outcomes will be analyzed using multivariable logistic regression with generalized estimating equations, including adjustments for baseline factors. The statistical test will be performed in a hierarchical order according to the succession of the objectives. The p-value will be corrected with the Bonferroni-Holm method. Potential effect modifiers considered are: sex, age, baseline treatment regime, and baseline comorbidities. All main analyses will follow an approach of intention-to-treat.

## Ethics and dissemination

### Ethical approval

The study was approved by the Ethical committee of the Region of Southern Denmark (Project-ID: S-20230015) in May 2023. The local ethical committee will be notified with a protocol amendment if any changes to the research project could potentially affect the participants.

The study is conducted in compliance with the Declaration of Helsinki II and registered at ClinicalTrials.gov in January 2024.

### Ethical considerations

Participation in this study involves an addition to the standard care that patients with CVD and DM2 already receive. The intervention in the CMC consists of a single visit with an in-depth and tailored review of the patient’s risk factors and potential optimization of treatment. The control group will continue to receive the standard care from their regular physician(s). Thus, the treatment should theoretically be equivalent, and we do not anticipate any associated risks from the intervention.

One ethical consideration is the risk of overburdening participants. This is addressed by conducting individual follow-up visits, typically by telephone. Participants are actively involved in the decision-making process for any new treatments and will be thoroughly informed of potential benefits, side effects and required monitoring beforehand. An individualized patient course is designed to ensure safety and provide appropriate support to participants who may feel overburdened. A secondary endpoint is to assess the patient-experienced symptoms and determine whether the intensive medication regimen could cause stress or anxiety for participants due to increased focus on their condition.

Another ethical consideration is ensuring equity and fairness. While we do not discriminate based on age, we have an exclusion criterion of a life expectancy less than 5 years to ensure the primary outcome can be assessed. Participants are not discriminated based on their socioeconomic status, however, some may chose not to participate if they feel they are overburdened, which could sometimes correlate with their socioeconomic status.

### Safety

Participants are monitored closely when new medication is prescribed. Potential side effects and tolerance to the medical treatment will be managed through individualized patient courses, which may include additional blood and urine samples, if applicable. Follow-up evaluation will be conducted by telephone consultations. Participants will continue to receive the standard care from their regular physician, who will be informed of any treatment changes made in the CMC through correspondence. Participants are advised to contact the project staff if they experience side effects or any issues related to the treatment initiated in the CMC.

All health examinations are considered harmless, non-invasive and involve minimal inconvenience and discomfort. The national patient insurance will cover the participants. No compensation or remuneration will be provided for participation in the study.

### Data management

The law regarding General Data Protection Regulation and the Data Protection Act will be followed in the management of personal information (i.e. journal or health information). Study information will be recorded, handled, and stored safely. Only persons with secrecy and connection to the study will access the data. Permission to process and store data was granted by the regional data protection agency at Odense University Hospital, and participant data will be stored using this electronic case-report form in REDCap.

### Dissemination

Scientific publications will be in accordance with the Vancouver recommendations. Positive, negative, or inconclusive results will be published in international peer-reviewed journals.

## Discussion

The purpose of this study is to establish an affordable Cardio-Metabolic Clinic that offers a multidisciplinary, specialized care with a specific emphasis on cardiovascular protection.

A major strength of the ProtecT-2-D study is the randomized controlled trial design. This design helps to minimize bias, and it provides a robust framework to assess the impact of the CMC compared to the standard of care without confounding influence. The intervention involves intensive diabetes management based on current guidelines, while the control group receives standard of care from their regular physician. If the study yields positive results and demonstrates reductions in CV morbidity and mortality, the CMC holds the potential to pave the way for integration of similar models into broader healthcare infrastructure. However, the single-center design conducted in Denmark may limit the applicability of the findings to diverse populations and healthcare settings internationally. Future research could explore the feasibility of similar clinic models in different geographical locations and healthcare contexts.

The concept of integrated cardio-metabolic clinics have traditionally involved high costs due to the collaboration between specialists in cardiology, endocrinology, and nephrology [22, 24, 25]. This multidisciplinary approach, although beneficial, have been questioned in terms of cost-effectiveness [26]. The EUROACTION trial [35] demonstrated the effectiveness of nurse-coordinated, multidisciplinary, family-based cardiovascular disease prevention programs, to significantly improve lifestyle changes and cardiovascular risk management in patients with coronary heart disease and those at high risk. Our CMC builds on these findings but aims to be more cost-effective. By employing supervised medical students, who are overseen by a cardiologist for 1–2 hours daily, and utilizing decision-making algorithms, we reduce the need for multiple specialists in routine cases, but ensure optimal, tailored treatment. This makes our approach more affordable while maintaining high standards of care.

We hypothesize that the CMC model will offer better management of cardiovascular and metabolic conditions compared to the traditional primary care, through the algorithm-driven treatment plans and specialist supervision. Decision-making algorithms provide consistent and standardized care by ensuring all patients receive evidence-based treatments according to the latest guidelines, reducing variability in care. A scoping review [36] of clinical decision support systems found them effective and safe in enhancing diabetes care, particularly beneficial for physicians with limited experience and patients who had limited access to medical resources.

The effectiveness of the interventions in the CMC is dependent on participants' compliance and adherence to recommended treatments and lifestyle modifications. Non-compliance or poor adherence could potentially diminish the impact of the CMC. The study is designed with only one initial visit, but with the flexibility to schedule individual visits when required. This design serves as both a limitation and a strength. While the study design may pose challenges in participants’ adherence to prescribed medications, on the other hand, it enhances the affordability and feasibility within the current organizational structure, thus making it a cost-effective solution.

## Perspectives

A future research perspective could be to extend the use of artificial intelligence (AI) into the clinic model. The use of AI technologies offer potential to optimize administrative tasks such as eligibility screening and appointment scheduling. Large language models could automate data entry and generate journal reports. Furthermore, AI could augment personalized treatment recommendations through decision-making algorithms based on patients’ medical history and lab results. In the present study, the use of AI could improve efficiency and reduce costs while also reducing the burden on the project staff. Another future research perspective could integrate the same multidisciplinary approach aimed at other high-risk populations. Diabetes is a major risk factor for development of CV disease, however, other chronic diseases are also known to exhibit a high CV risk. For instance, rheumatologic conditions such as systemic lupus erythematosus, rheumatoid- and psoriasis arthritis, are associated with increased risk of CV events independent of traditional risk factors, and they might also benefit from a similar clinic concept [37].

## Conclusion

In conclusion, the Cardio-Metabolic Clinic represents a pioneering, multidisciplinary approach to diabetes management that aims to improve patient outcomes by reducing the cardiovascular disease burden. Establishing the efficacy and feasibility of the Cardio-metabolic Clinic may facilitate the integration of similar clinics into broader healthcare systems, which potentially could enhance cardiovascular health in patients with type 2 diabetes and cardiovascular disease.

### Supplementary Information

Protocol - Cardiovascular Protection in Patients with Type 2 Diabetes and Established Heart or Vascular Disease - The Cardio-Metabolic Clinic (This document contains the full protocol for the study). Supplementary Material 1

SPIRIT-Outcomes 2022 Checklist (This document contains the completed checklist for the SPIRIT 2013 and SPIRIT Outcomes 2022 guidelines). Supplementary Material 2

## Data Availability

Not applicable.

## Abbreviations

T2D: Type 2 diabetes
CV: Cardiovascular
ACEi: Angiotensin-converting-enzyme inhibitor
SGLT2i: Sodium-glucose co-transporter 2 inhibitor
GLP1-RA: Glucagon-like peptide 1 receptor agonist
CMC: Cardio-Metabolic Clinic
PAD: Peripheral arterial disease
HF: Heart failure
CVD: Cardiovascular disease
AI: Artificial intelligence
