# Supplementary material for: ProtecT-2-D trial protocol: cardiovascular protection in patients with type 2 diabetes and established heart and/or vascular disease at a cardio-metabolic clinic—a randomized controlled trial
Source: Cardiovasc Diabetol. 2024 Jul 8;23:241. doi: 10.1186/s12933-024-02340-w (PMC11232310; doi:10.1186/s12933-024-02340-w)
Supplement: Supplementary file 1 — Protocol - Cardiovascular Protection in Patients with Type 2 Diabetes and Established Heart or Vascular Disease - The Cardio-Metabolic Clinic (This document contains the full protocol for the study). Supplementary Material 1 [file 12933_2024_2340_MOESM1_ESM.docx]

PROTOCOL

Cardiovascular Protection in Patients with Type 2 Diabetes and Established Heart or Vascular Disease - The Cardio-Metabolic Clinic

ProtecT-2-D STUDY

*Trial registration identification:*

ClinicalTrials.gov: NCT06203860. 11.Jan.2024

Ethics committee approval: S-20230015. 23.May.2023.

Roles and responsibilities:

*Contributors:* Katrine S. Overgaard ^1,2^, Roda A. Mohamed^1,2^, Thomas R. Andersen^1,2^, Jess Lambrechtsen^1,2^, Kenneth Egstrup^1,2^ Søren Auscher^1,2^

Affiliations:

^1.^ Cardiovascular Research Unit, Odense University Hospital Svendborg, Baagøes Allé 15, 5700 Svendborg, Denmark
^2.^ Department of Clinical Research, [Faculty of Health Sciences](https://portal.findresearcher.sdu.dk/en/organisations/det-sundhedsvidenskabelige-fakultet), [University of Southern Denmark](https://portal.findresearcher.sdu.dk/en/organisations/syddansk-universitet), Campusvej 55, 5230 Odense M, Denmark

Principal investigator: Søren Auscher, M.D, Ph.D

Cardiovascular Research Unit, Odense University Hospital – Svendborg

Baagoes Allé 15, Entrance 51c. DK-5700 Svendborg

Telephone: +45 6320 2402

Email: [Soeren.Auscher@rsyd.dk](mailto:Soeren.Auscher@rsyd.dk)

**Authors’ contributions:**

KE and SA conceived the idea for the study. *SA* is the principal investigator. KE and SA designed the trial with inputs from KSO, RAM, JL and TRA. KE is grant holder. RAM wrote the statistical analysis plan. TRA developed the decision-making algorithm. *KSO* were in charge of protocol writing and trial registrations. All authors contributed to refinement of the study protocol and approved the final manuscript.

*Sponsor: Odense University Hospital*

This funding source had no role in the design of this study and will not have any role during its execution, analyses, interpretation of the data, or decision to submit results.

**WHO trial registration data set items**

| **DATA CATEGORY** | **INFORMATION** |
| --- | --- |
| **Primary registry and trial identifying number** | ClinicalTrials.gov NCT06203860 |
| **Date of registration in primary registry** | 11, January 2024 |
| **Secondary identifying numbers** | S-20230015 |
| **Source(s) of monetary or material support** | Odense University Hospital |
| **Primary sponsor** | Odense University Hospital |
| **Contact for public queries** | Søren Auscher, M.D, PhD, [+45 6320 2402] [[Soeren.Auscher@rsyd.dk](mailto:Soeren.Auscher@rsyd.dk)] |
| **Contact for scientific queries** | Søren Auscher, M.D, PhD, Cardiovascular Research Unit, Odense University Hospital – Svendborg |
| **Public title** | The Cardio-Metabolic Clinic (ProtecT-2-D) |
| **Scientific title** | Cardiovascular Protection in Patients with Type 2 Diabetes and Established Heart or Vascular Disease - The Cardio-Metabolic Clinic |
| **Countries of recruitment** | Denmark |
| **Health condition(s) or problem(s) studied** | Type 2 Diabetes, Heart disease, Vascular disease, Cardiovascular disease, organizational structure |
| **Intervention(s)** | Active comparator: Cardio-Metabolic Clinic  Placebo comparator: Standard of care |
| **Key inclusion and exclusion criteria** | Ages eligible for study: ≥18 years Sexes eligible for study: both Accepts healthy volunteers: no  Inclusion criteria:  Diagnosed with type 2 diabetes,  Established heart or vascular disease defined as: (1) Atherosclerotic disease (acute or chronic coronary syndrome, stroke, peripheral arterial disease, imaging proved ischemic heart disease),  (2) Heart failure,  (3) Atrial fibrillation,  (4) Valvular heart disease,  (5) Severe hypertension.  Exclusion criteria:  Short life expectancy, another diabetes type, participation in other interfering clinical trials. |
| **Study type** | Interventional Allocation: randomized Intervention model: 2:1 parallel assignment  Masking: no  Primary purpose: prevention |
| **Date of first enrolment** | 01. Jan. 2024. |
| **Target sample size** | 1500 |
| **Recruitment status** | Recruiting |
| **Primary outcome** | The time to the first occurrence of major adverse cardiovascular event (MACE), a composite endpoint consisting of: Cardiovascular death, non-fatal acute myocardial infarction, non-fatal stroke, or hospitalization for heart failure (time frame: 5 years) |
| **Key secondary outcomes** | The time to the first occurrence of one of the single components of the primary outcome, and a combination to assess the total symptom burden. Assessment of microvascular complications, protocol driven medical changes and a cost-effectiveness analysis. |

Table of contents

[Background 6](#_Toc170215081)

[Hypothesis 7](#_Toc170215082)

[Aim 7](#_Toc170215083)

[Study design 7](#_Toc170215084)

[Methods 7](#_Toc170215085)

[Study setting 7](#_Toc170215086)

[Study population 7](#_Toc170215087)

[Eligibility criteria 7](#_Toc170215088)

[Screening 9](#_Toc170215089)

[Informed consent procedures 9](#_Toc170215090)

[Baseline visit 10](#_Toc170215091)

[Randomization 11](#_Toc170215092)

[Interventions – the Cardio-Metabolic Clinic 11](#_Toc170215093)

[Treatment algorithm: 13](#_Toc170215094)

[• Dyslipidemia: 13](#_Toc170215095)

[• Hypertension: 13](#_Toc170215096)

[• Thrombosis prophylaxis 13](#_Toc170215097)

[• Diabetes 14](#_Toc170215098)

[• Microvascular complications 14](#_Toc170215099)

[• Evaluation of prescription of SGLT2 inhibitor and/or GLP-1 receptor agonist: 14](#_Toc170215100)

[• Lifestyle: 15](#_Toc170215101)

[• Peripheral artery disease/foot ulcer: 15](#_Toc170215102)

[• Vaccinations: 15](#_Toc170215103)

[Examinations 15](#_Toc170215104)

[Follow-up visits 16](#_Toc170215105)

[Discontinuation: 17](#_Toc170215106)

[Timeline 17](#_Toc170215107)

[Outcomes 18](#_Toc170215108)

[Statistics 22](#_Toc170215109)

[Ethics and dissemination 23](#_Toc170215110)

[Financing 24](#_Toc170215111)

[References 25](#_Toc170215112)

# Background

The number of patients with type 2 diabetes (T2D) has more than tripled during the last twenty-five years and every 20^th^ person in the Danish population is currently living with T2D (1). The risk of developing cardiovascular (CV) disease or premature death is 2-4 times higher in patients with T2D compared with the general population (2). T2D is a systemic disease that is associated with both macro- and microvascular complications, and is often accompanied by other metabolic disorders like hypertension, dyslipidemia, obesity, and metabolic dysfunction-associated steatohepatitis (MASH). A recent American study (3) showed that half of T2D patients suffered from at least three other cardio-renal-metabolic conditions. Furthermore, patients with T2D have an increased risk of developing diabetic cardiomyopathy and heart failure (4).

The STENO-2-Study (5) demonstrated that multifactorial intervention on CV risk factors in patients with T2D significantly reduced mortality (6), and treatment with statins, angiotensin-converting-enzyme (ACE) inhibitors, and anti-glycemic drugs are the cornerstone in the management of patients with T2D (7, 8). Similarly, the prospective RAMP-study with more than 50.000 participants demonstrated a substantial reduction in CV risk (56.6 %), microvascular complications (11.9 %), and mortality (66.1 %) through multifactorial diabetes and risk factor intervention compared to the standard of care (9, 10).

Recently, new medication has emerged in the field of anti-diabetic treatment and has revolutionized the management of CV risk in patients with T2D. Treatment with sodium-glucose co-transporter 2 (SGLT2) inhibitors and glucagon-like peptide 1 (GLP-1) analogs have been a “game changer” in diabetes treatment and have been shown to reduce CV mortality, cardiovascular events and to be very effective in patients with heart failure and reduced kidney function (11-19). Despite these effective medical treatments, patients with T2D still have an increased risk of cardiovascular death and development of heart failure(2). Furthermore, it is well known that many patients with T2D struggle to reach their target goals for blood sugar levels and CV risk factors (20). Traditionally, the healthcare system focuses on single-disease management. Patients with T2D are managed in specialty-focused outpatient clinics resulting in fragmented care, inadequate treatment, higher costs, and worse CV outcome (21, 22). Therefore, there is a desperate need for a more multidisciplinary and thorough assessment of patients with T2D, and a closer collaboration between endocrinologists, nephrologists and cardiologists in the disease management.

We propose establishing a Cardio-Metabolic Clinic, providing comprehensive assessment and specialized care following current diabetes guidelines. The concept of such clinics have previously been emphasized, often involving collaboration between specialists in cardiology and endocrinology, resulting in high costs (21, 23). Additionally, a recent analysis questioned the cost-effectiveness of primary care based cardio-metabolic risk prevention programs (24). Our goal is to develop an affordable and resource-efficient clinic model. This involves implementing a decision-making algorithm to guide patient care, with medical students or nurses providing the daily patient contact under supervision of a cardiologist.
The primary aim of this randomized controlled trial is to investigate whether a Cardio-Metabolic Clinic is superior to the standard of care in reducing cardiovascular morbidity and mortality in patients with type 2 diabetes and established cardiovascular disease.

## Hypothesis

In patients with T2D and cardiovascular disease, a systematic, specialized multidisciplinary approach in a Cardio-Metabolic Clinic, will result in better management of diabetes and reduced cardiovascular morbidity and mortality.

Aim
This prospective, randomized controlled trial is designed to explore the benefits of a systematic, specialized multidisciplinary approach in a Cardio-Metabolic Clinic in patients with T2D and established cardiovascular disease.

Study design
The study is a single-center, open label, prospective, randomized controlled trial examining the efficacy of a specialized, multidisciplinary approach in patients with T2D and established CV disease (CVD) compared with usual care.

# Methods

Study setting
The study will be carried out at the Cardiovascular Research Unit at OUH, Svendborg Hospital, Denmark.

Study population
Patients with T2D and established CVD, referred from general practices or seen in the outpatient clinic of Cardiology or Endocrinology at Svendborg Hospital, will undergo screening for potential inclusion in the study.

Eligibility criteria
Inclusion criteria:

- >18 years.
- Capable of giving written informed consent.
- Established diagnosis of T2D [26].
- Established heart or vascular disease, defined as either:

1. Atherosclerotic disease defined as:
   i) Prior acute coronary syndrome [27].

ii) Chronic coronary syndrome defined as the combination of: Angina pectoris AND coronary atherosclerosis assessed with either coronary CT angiography or myocardial-scintigraphy or coronary angiography AND treatment with statins and/or acetylsalicylic acid [28].
iii) Stroke [29].
iv) Peripheral arterial disease (PAD) defined as: Claudication intermittence in combination with pathological ankle brachial index AND/OR vascular PAD surgery AND/OR ischemic amputation [30]. v) Ischemic heart disease defined by one of the following criteria: a) Myocardial-scintigraphy: >10% reversibility OR b) Coronary CT angiography: Coronary Artery Calcium-score >100.

1. Heart failure (HF): HF with reduced ejection fraction, HF with Mildly reduced ejection fraction, HF with preserved ejection fraction [31].
2. Atrial fibrillation and/or flutter, including paroxysmal, persistent and chronic disease [32].
3. Valvular heart disease (which requires control in outpatient clinic of cardiology), such as aortic valve stenosis, mitral valve insufficiency, and patients with aortic dilatation.
4. Hypertension treated with at least three antihypertensive drugs.

Exclusion criteria:

1. Life expectancy less than 5 years for any reason.
2. Type 1 Diabetes Mellitus [26].
3. Participation in another clinical trial with an investigational product or device that could interfere with the primary and/or secondary endpoints of this study.

## Screening

Participants will be selected from patients with T2D and established CVD, referred from general practices or seen in the outpatient clinic of Cardiology or Endocrinology at Svendborg Hospital between January 2024 and January 2027 (see figure 1). If applicable, by the in- and exclusion criteria, patients will be contacted by a letter and the study staff will inform the patients of the study. If patients are interested in participation, possible dates for a visit will be arranged.

Informed consent procedures
Eligible participants will receive written material (participant information) by a letter in the e-Boks system before the first visit. On the first visit, patients are given oral information regarding the study and informed consent will be obtained before conducting any study-specific procedures.

**Figure 1: Screening and management program in the Cardio-Metabolic Clinic**

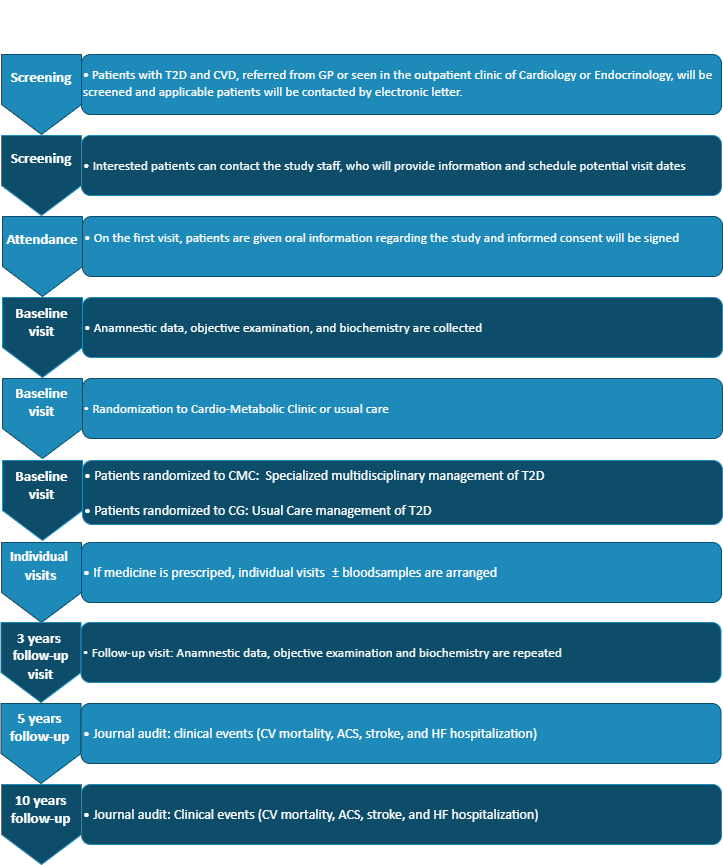


*Abbreviations: T2D= Type 2 diabetes, CVD= Cardiovascular disease, GP= General practice, CMC= Cardio-Metabolic clinic, CG=Control group, CV= Cardiovascular, ACS= Acute coronary syndrome, HF= Heart failure.*

Baseline visit
All participant data, including detailed medical histories, will be digitally captured in the electronic-Case Report Form (REDCap). The assessments will encompass: Duration and diabetes-related complications, demographic data (gender, age), cardiovascular disease (including heart failure, ischemic heart disease, atrial fibrillation, valve diseases, peripheral arterial disease, stroke), risk factor status (including hypertension, hypercholesterolemia, smoking habits, alcohol consumption, familiar disposition to ischemic disease), and a detailed record of pharmacological treatments including vaccinations.

Verification of diabetes-related complication such as retinopathy, nephropathy, and neuropathy will be conducted via the Danish Adult Diabetes Database.
Physical examination will be executed, including anthropometric measurements (height, weight, and hip/waist ratio), and blood pressure will be measured after a 10-15 minutes rest period. Additionally, evaluations will include an inspection for diabetic foot ulcers and measurement of the ankle/brachial index (ABI). Electrocardiographic monitoring will be performed.

Biochemistry: Blood and urine samples will be collected. Albuminuria will be evaluated from a spot urine sample. Patients with high levels of the biomarker FIB 4 will have a Fibro-scan performed.

Randomization
Participants, which fulfill the inclusion and exclusion criteria, will be randomized to one of the following programs in a ratio of 2:1. Randomization will be performed using the randomization module available in the REDCap system.

1. Cardio-metabolic group (CMC): Specialized multidisciplinary management of T2D in a Cardio-Metabolic Clinic with a focus on the management of cardiovascular risk factors and assessment of all micro- and macrovascular complications.

2. Control group (CG): Standard of care management of T2D. This consists of a collaboration between the general practitioner and/or endocrinology outpatient clinic and/or cardiology outpatient clinic.

## Interventions – the Cardio-Metabolic Clinic

#### Organization in the Cardio-Metabolic clinic

The Cardio-metabolic clinic is based on a low-cost model and is organized in three different layers centered on the patient. The innermost layer consists of medical students or specialized cardio-metabolic nurses, who have the daily contact with the patients in the Cardio-metabolic Clinic. Patient´s medical history and data from the baseline visit are entered into the Electronic Case-Report Form (REDCap). When Patients are randomized to the intervention arm a decision-making algorithm in the REDCap-system will be activated ensuring that patients receive the most optimal and tailored medical treatment according to the latest guidelines and recommendations for diabetes management. The second layer consists of a cardiologist who the medical students or cardio-metabolic nurses will review the patients risk profile and algorithm-recommended treatment with. If the cardiologist needs further counselling for patient management, the third layer will be contacted. The third layer consists of an endocrinologist, a nephrologist and a hepatologist and this multidisciplinary collaboration will ensure the most optimal diabetes management in challenging cases.

#### Interventions in the Cardio-Metabolic clinic:

Participants in the CMC will follow a decision-making treatment algorithm in the REDCap-system. The treatment algorithm is coded in accordance to the most recent guidelines and recommendations for diabetes management and CVD (32). Consequently, there may be modifications over time. Participants will be categorized into various cardiovascular risk groups using the SCORE2-Diabetes (Figure 2) (32) and will receive interventions targeting nine specific areas (Figure 3); dyslipidemia, hypertension, thrombosis prophylaxis, diabetes management, assessment of microvascular complications, evaluation of SGLT2i and/or GLP1-RA, lifestyle modifications, peripheral artery disease, and vaccinations. The CMC intervention is considered complete when a participant is fully titrated to the targeted or highest tolerated dose in all suggested treatment areas. Endpoint assessments will begin once intervention is completed.


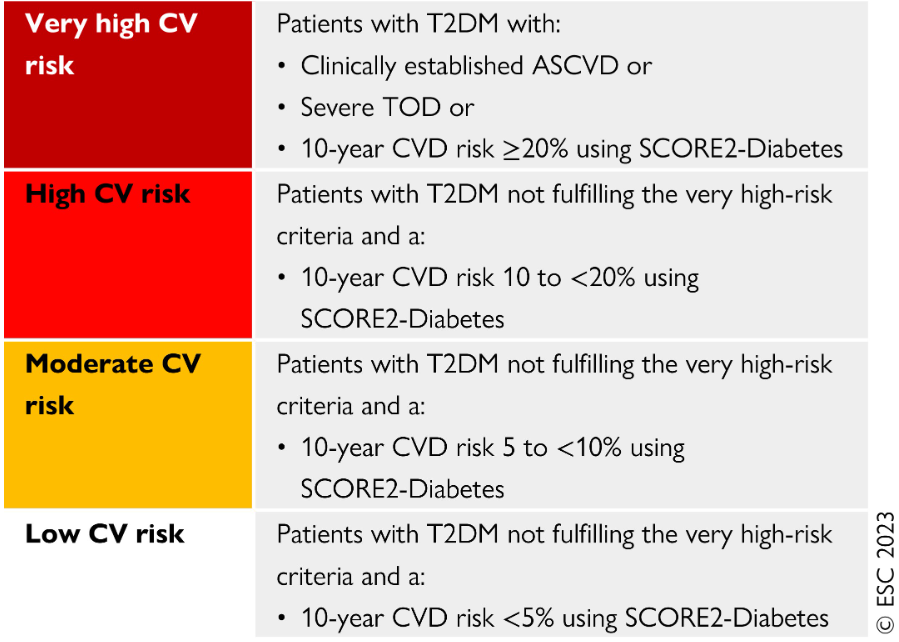
**Figure 2. SCORE2-Diabetes. Cardiovascular risk categories in type 2 diabetes**

*Burrowed from* 2023 ESC Guidelines for the management of cardiovascular disease in patients with diabetes (32).  *Abbreviations: CV= Cardiovascular, T2D= Type 2 diabetes, ASCVD= Atherosclerotic cardiovascular disease, TOD= Target organ damage, CVD= Cardiovascular disease.*

## Treatment algorithm:

**Figure 3. Overview of the treatment algorithm in the CMC**

**
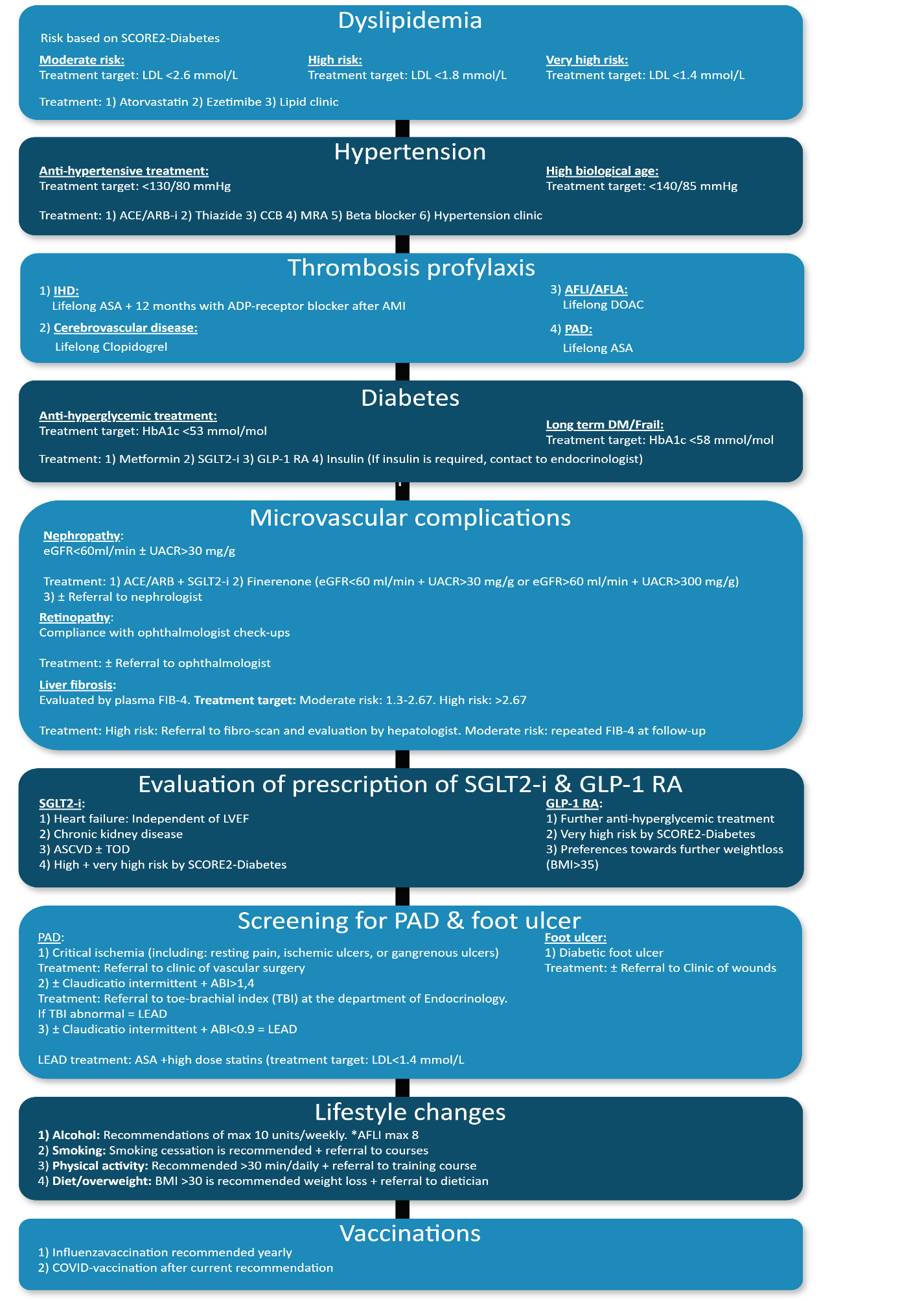
**

- Dyslipidemia:
  Dyslipidemia is assessed by LDL status in blood samples. Patients are classified according to table 1 (32). Patients with moderate CV risk; will be recommended a LDL <2.6 mmol/L.
  Patients with high CV risk; will be recommended a LDL <1.8 mmol/L.
  Patients with very high CV risk; will be recommended a LDL <1.4 mmol/L (32).

If LDL exceed these thresholds, the treatment algorithm will be according to the following order: 1) High dose Atorvastatin, 2) Ezetimibe, 3) Referral to lipid-clinic.

- Hypertension:
  Blood pressure is measured in the examination, patients with a blood pressure >130/80 mmHg are instructed in measurement of blood pressure at home 3 times a day for three days (32). If the mean home blood pressure is >130/80 mmHg, treatment algorithm will be according to the following order:

1) ACE-inhibitor or angiotensin II receptor blocker (ARB), 2) thiazide, 3) calcium antagonist, 4) mineralocorticoid (MRA), and 5) beta blocker. If aforementioned is already supplied 6) Referral to hypertension-clinic.

In patients with a high biological age blood pressure <140/85 mmHg is accepted. Blood pressure >140/85 mmHg the above-mentioned treatment algorithm will be followed (32).

- Thrombosis prophylaxis:
  Patients with ischemic heart disease are recommended lifelong acetylsalicylic acid.
  If patients have had a new acute myocardium infarction, they will be prescribed 12 months of ADP-receptor blocker (32).

Patients with cerebrovascular disease are recommended lifelong clopidogrel treatment(32) . Patients with periphery arterial disease are recommended lifelong ASA treatment (32).

Patients with atrial fibrillation/flutter are recommended lifelong direct oral anti-coagulant (DOAC) treatment (32).

When two antithrombotic drugs are used, proton pump inhibitors are recommended to prevent gastrointestinal bleeding(32).

- Diabetes: Hyperglycemia is assessed by measurement of HbA1c. If HbA1c > 53 mmol/mol addition of antidiabetic treatment is required (32). Exceptionally, in patients with a long diabetes duration (>10 years), high age (> 65 years), and episodes with hypoglycemia, a HbA1c <58 mmol/mol will be accepted (33, 34). The standard treatment algorithm will be according to the following order: 1) Biguanides 2) SGLT2 inhibitor, 3) GLP-1 receptor agonists 4) Insulin. In patients with atherosclerotic cardiovascular (ASCVD) treatment with a SGLT2 inhibitor and/or GLP-1 receptor agonist will be first. If insulin treatment is required, an endocrinologist will be contacted.
- Microvascular complications: - Nephropathy: Defined by reduced eGFR <60 ml/min and/or increased urine albumin/creatinine ratio (UACR) >30mg/g. Patients will be treated with maximum tolerable dosis of 1) ACE-inhibitor/ARB and SGLT2 inhibitor. 2) Finerenone, if 1) is fulfilled and either: eGFR>60 ml/min + UACR 300 mg/g or eGFR< 60 ml/min + UACR >30 mg/g (32).
  If further assessment of nephropathy is required, a nephrologist will be contacted.

- Retinopathy: Compliance with ophthalmologist check-ups. If non-obedient referral to ophthalmologist check-up.

- Evaluation of liver fibrosis: Systematic screening using serological test with FIB-4 will be performed at baseline and at the 3 years follow-up visit. Patients with moderate risk (FIB-4: 1.3 - 2.67) at baseline and at 3 years follow-up and patients with high risk (FIB-4: > 2,67) will be referred to the department of gastroenterology for a Fibro-scan. In case of suspicion of fibrosis or cirrhosis an individual risk assessment will be made by a hepatologist.

- Evaluation of prescription of SGLT2 inhibitor and/or GLP-1 receptor agonist:
  SGLT2 inhibitor will be prescribed if one of the following are present (32) :

1. Heart failure (regardless of ejection fraction)

2. Chronic kidney disease

3. Atherosclerotic heart disease

4. In patients with a high or very high CV risk (table 1).

GLP-1 receptor agonists will be prescribed if one of the following are present (32):

1. If further hyperglycemic control is needed on top of treatment with Biguanid and/or SGLT2-i.
2. In patients with a very high CV risk (table 1).

3. Preferences towards further weight loss (BMI>35).

- Lifestyle:
  Medical history regarding lifestyle and behavior is obtained, and recommendations for lifestyle changes will be provided (32). Alcohol consumption is recommended to a max of 10 units weekly (35), and if diagnosed with atrial fibrillation/flutter max of 8 units weekly (36). If patients are actively smoking, smoking cessation is recommended. Patients are motivated, and if interested referred to cessation courses (32). A Mediterranean diet supplemented with olive oil and/or nuts reduces the incidence of major CV events in patients with CVD (32). Patients are recommended physical activity of at least 30 minutes daily. If interested, patients can be referred to training sessions. Patients with a BMI >30 is recommended for weight loss and can be referred to a dietician or treatment with GLP-1 analogs may be considered (32).

### Peripheral artery disease/foot ulcer:

1) Patients with critical ischemia (including: resting pain, ischemic ulcers, or gangrenous ulcers) will be referred to the outpatient clinic of vascular surgery (32, 37).

2) Patients with or without symptoms of intermittent claudicatio and ABI>1,4 will be referred to examination with toe-brachial index (TBI) at the department of Endocrinology. If TBI is abnormal, patients will be diagnosed with lower extremity artery disease (LEAD), and treatment with ASA and high dose statins therapy will be initiated. Target LDL level <1.4 mmol/L (32, 37).

3) Patients with or without symptoms of intermittent claudicatio and ABI<0.9 are diagnosed with lower extremity artery disease (LEAD), and treatment with ASA and high dose statins therapy will be initiated. Target LDL level <1.4 mmol/L (32, 37).

4) Patients with diabetic foot ulcers will be referred to the outpatient clinic for wounds (32, 37).

5) Patients with abnormal monofilament test and symptoms of neuropathy will be referred to the department of endocrinology (32).

- Vaccinations:
  Anamnesis regarding vaccination status is obtained, and recommendations of yearly influenza vaccination (38). COVID vaccinations (39) after current local guidelines.

Examinations
1) Blood samples will be drawn at the baseline visit and at the three years follow-up visit. Approximately 80 ml of blood will be collected. Blood samples will be analyzed for; Hematology: Hemoglobin and leucocytes.
Lipid profiles: Total cholesterol, high-density lipoprotein (HDL), low-density lipoprotein (LDL), and triglycerides. Diabetes status: Glycated haemoglobin (HbA1c). Liver function: Alanine transaminase (ALAT), alkaline phosphatase, and gamma-glutamyl transferase (GGT), lactate dehydrogenase (LDH), and FIB-4 index for liver fibrosis assessment. Cardiac markers: N-terminal-pro b-type natriuretic peptide (NT-proBNP) and troponin T. Electrolytes: Sodium and potassium. Renal function: Creatinine and estimated glomerular filtration rate. Inflammatory marker: C-reactive protein (CRP).

2) Urine samples will be collected at the baseline visit and at the 3 year follow-up visit and albuminuria will be evaluated. This will include measurement of urine albumin and creatinine to calculate the albumin creatinine ratio (UACR) from a spot urine sample.

3) Blood pressure measurement: Blood pressure will be measured at baseline and at the 3 year follow-up visit. If blood pressure is above 130/80 mmHg after 10 minutes the CMC group will measure blood pressure at home (3 times daily for 3 days) and be treated according to the Cardio-metabolic treatment algorithm. Patients in the control group will be advised to contact the general practitioner (GP) in case of blood pressure above 130/80 mmHg.

4) Ankle-brachial index (ABI): ABI will be assessed at the baseline visit and at the 3 year follow-up visit. During the procedure, patients will be instructed to lie down in a supine position and rest for a period of 10 minutes. Blood pressure measurements will be performed in both the brachial arteries and the dorsalis pedis arteries using a blood pressure cuff and a Doppler ultrasound devise. ABI will hereby be calculated.

5) Monofilament test: will be performed at the baseline visit and at the 3 year follow-up visit. This test involves applying a 10-g monofilament at the distal plantar aspect of both great toes and metatarsal joints to assess pressure sensation.

5) Transient Elastography (Fibro-scan): Patients will be evaluated for liver fibrosis using serological test with FIB-4 at baseline and at 3 year follow-up visit. Patients with high risk (FIB-4: > 2,67) or moderate risk (FIB-4: 1.3 - 2.67) at baseline and at 3 year follow-up will be referred to the department of gastroenterology for a fibro-scan. In case of suspicion of fibrosis or cirrhosis an individual risk assessment will be made by a hepatologist.

## Follow-up visits

**Individual visits:**If a new treatment is prescribed that causes need for further monitoring or evaluation, individual visits as either telephone consultations or attendances will be arranged. In some cases, additional blood samples, urine samples will be collected and evaluated. Efforts will be made to keep participants on study intervention until they are completed in the CMC intervention.

**Three-year follow-up visit:**
At the three-year follow-up, all participants will undergo the same evaluation program as outlined during the baseline visit. They will be evaluated for clinical events related to the primary and secondary outcomes. Blood- and urine samples will be collected from all participants.

Efforts will be made to ensure that participants attend and complete the three years follow up visit. Participants will receive their appointment for this visit during the baseline visit. Additionally, they will be sent an electronic letter one month before the scheduled follow-up and will be reminded by a telephone call from the study staff.

**Five year follow-up:**
A journal audit and look-up in the Danish Health Data Authority will be performed to examine the number of clinical events according to the primary and secondary endpoints.

**Ten year follow-up**:
A journal audit and look-up in the Danish Health Data Authority will be performed to examine the number of clinical events according to the primary and secondary endpoints.

## Discontinuation:

Participants will be followed for the complete duration of the study regardless of their adherence to the study intervention. Any component of the study intervention may be discontinued at the discretion of either the participant or the study staff due to safety, behavioral or compliance reasons.

Withdrawal of consent: Participant may withdraw consent at any time upon request. Those considering withdrawal should be offered the option of flexible participation, if feasible. If a participant withdraws consent, any data collected before such a withdrawal of consent may still be used for study purposes. Participants who withdraw will be included as intention-to-treat in the statistical analysis once the study ends.

Lost to follow up: If participants fail to attend and complete the three years follow up visit, they will be considered lost to follow-up for the analysis of the secondary outcomes that requires attendance. However, these participants will still be included in the primary and secondary prognostic outcomes.

Timeline
The study started in January 2024, with a four-year inclusion period that will continue until January 2028. After three years, all patients will have a follow-up visit and baseline assessments will be repeated. Clinical events will be assessed at 5- and 10-years follow-up by journal audits and by look-up in the Danish Health Data Authority. Thus, the 5 years follow-up will be approximately in January 2031, and 10 years follow-up in January 2036 (figure 4).

**Figure 4 - Timeline for the study**


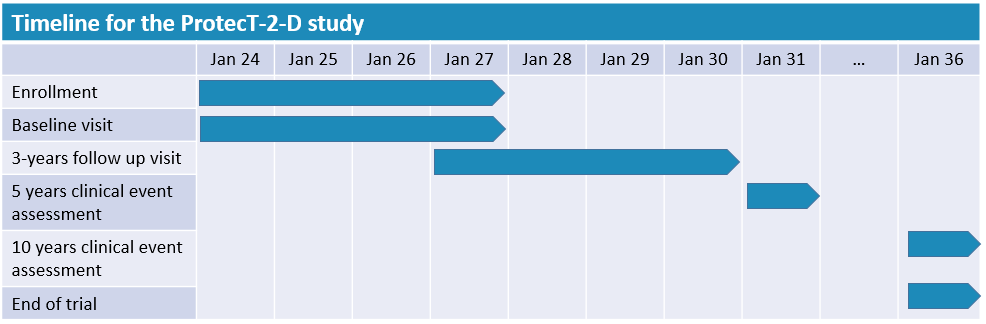


Outcomes The primary outcome is the time to the first occurrence of a composite of CV deaths, non-fatal myocardial infarction, non-fatal stroke, and hospitalization for heart failure.

The secondary outcomes are the time to the first occurrence of one of the single components of the primary outcome, and a combination of the single components of the primary outcome to assess the total symptom burden. Additional secondary outcomes will be the assessment of microvascular complications, protocol driven medical changes and a cost-effectiveness analysis of the Cardio Metabolic Clinic. Primary and secondary outcomes and endpoints are shown in the following table:

| **Primary outcomes** | **Primary endpoints** |
| --- | --- |
| To examine whether patients with type 2 Diabetes (T2D) receiving optimal diabetes care in a Cardio-Metabolic Clinic are superior compared to usual care in reducing; incidence of cardiovascular (CV) death, non-fatal acute myocardial infarction (AMI), non-fatal stroke, and hospitalization for heart failure (HF) | Time to the first occurrence of major adverse cardiovascular event (MACE), a composite endpoint consisting of:   - CV death - Non-fatal AMI - Non-fatal stroke - Hospitalization for HF |
| Units of measure: Measured in days. | Time frame: From baseline to 5 years of follow-up (FU). |

| **Secondary outcomes** | **Secondary endpoints** |
| --- | --- |
| To examine whether patients with type 2 Diabetes (T2D) receiving optimal diabetes care in a Cardio-Metabolic Clinic are superior compared to usual care in reducing; incidence of cardiovascular (CV) death, non-fatal acute myocardial infarction (AMI), non-fatal stroke, and hospitalization for heart failure (HF) | Time to the first occurrence of MACE, a composite endpoint consisting of:   - CV death - Non-fatal AMI - Non-fatal stroke - Hospitalization for HF |
| Units of measure: Measured in days. | Time frame: From baseline to 10 years FU. |
| To compare the individual component, CV death, of the primary endpoint in patients receiving optimal diabetes care in a Cardio-Metabolic Clinic versus usual care. | Time to the first occurrence of:   - CV death   (Presumed cardiac condition leading to death. Including: AMI, venous thromboembolic event, malignant arrhythmia, cardiogenic shock, fatal stroke and aorta dissection) |
| Units of measure: Measured in days. | Time frame: From baseline to 5 + 10 years of FU. |
| To compare the individual component, non-fatal AMI, of the primary endpoint in patients receiving optimal diabetes care in a Cardio-Metabolic Clinic versus usual care. | Time to the first occurrence of:   - Non-fatal AMI   Including: ST-elevation myocardial infarction, non-ST-elevation myocardial infarction |
| Units of measure: Measured in days. | Time frame: From baseline to 5 + 10 years of FU. |
| To compare the individual component, non-fatal stroke, of the primary endpoint in patients receiving optimal diabetes care in a Cardio-Metabolic Clinic versus usual care | Time to the first occurrence of:   - Non-fatal stroke   Including: Thromboembolic or undetermined |
| Units of measure: Measured in days. | Time frame: From baseline to 5 + 10 years of FU. |
| To compare the individual component, de novo HF and/or HF hospitalization, of the primary endpoint in patients receiving optimal diabetes care in a Cardio-Metabolic Clinic versus usual care | Time to the first occurrence of:   - De Novo Heart Failure - Heart Failure hospitalization |
| Units of measure: Measured in days. | Time frame: From baseline to 5 + 10 years of FU. |
| To assess the total symptom burden by a combination of the single component of the primary endpoints in patients receiving optimal diabetes care in a Cardio-Metabolic Clinic versus usual care | Number of overall symptom burden determined by summing the occurrences of:   - CV death - Non-fatal AMI - Non-fatal stroke - Hospitalization for HF |
| Units of measure: Measured in count of events. | Time frame: From baseline to 5 + 10 years of FU. |
| To compare the occurrence of microvascular complication, retinopathy, in patients receiving optimal diabetes care in a Cardio-Metabolic Clinic versus usual care | - Change in diabetic retinopathy stage based on eye examination (Fundoscopy) |
| Units of measure: Measured in ratio to baseline. | Time frame: From baseline to 3 years of FU. |
| To compare the occurrence of microvascular complication, nephropathy, in patients receiving optimal diabetes care in a Cardio-Metabolic Clinic versus usual care | - Change in estimated Glomerular Filtration Rate (eGFR)   Creatinine-based. |
| Units of measure: Measured in in ratio to baseline [mL/min/1.73 m^2]. | Time Frame: From baseline to 3 years of FU. |
| To compare the occurrence of microvascular complication, nephropathy, in patients receiving optimal diabetes care in a Cardio-Metabolic Clinic versus usual care | - Change in urinary albumin-to-creatinine ratio (UACR) |
| Units of measure: Measured in ratio to baseline. | Time Frame: From baseline to 3 years of FU. |
| To compare the occurrence of microvascular complication, nephropathy, in patients receiving optimal diabetes care in a Cardio-Metabolic Clinic versus usual care | - Change in Chronic Kidney Disease (CKD) stage   Calculated by eGFR and albuminuria. |
| Units of measure: Measured in ratio to baseline. | Time Frame: From baseline to 3 years of FU. |
| To compare the occurrence of microvascular complication, nephropathy, in patients receiving optimal diabetes care in a Cardio-Metabolic Clinic versus usual care | Time to first occurrence of a composite CKD endpoint consisting of:   - a decline in eGFR of more than 50%, - onset of end-stage kidney disease (dialysis, eGFR<15, kidney transplantation) - death from renal or CV causes |
| Units of measure: Measured in count of events. | Time Frame: From baseline to 3 years of FU. |
| To compare the occurrence of microvascular complication, liver fibrosis, in patients receiving optimal diabetes care in a Cardio-Metabolic Clinic versus usual care | - Change in fibrosis-4 (FIB-4).   FIB-4 is calculated using age, aspartate aminotransferase (ASAT), alanine aminotransferase (ALAT), and platelet count. |
| Units of measure: Measured in ratio to baseline. | Time Frame: From baseline to 3 years of FU. |
| To compare the occurrence of microvascular complication, liver fibrosis, in in high-risk patients receiving optimal diabetes care in a Cardio-Metabolic Clinic versus usual care | - Change in degree of liver fibrosis through a Fibro-scan |
| Units of measure: Measured in count of events. | Time Frame: From baseline to 3 years of FU. |
| To compare the occurrence of macrovascular complications, peripheral artery disease (PAD) and/or ischemic heart disease (IHD), in patients receiving optimal diabetes care in a Cardio-Metabolic Clinic versus usual care | Time to first occurrence of a composite macrovasucular endpoint comprising:   - new diagnosis of lower extremity arterial disease (LEAD), - new/progression of foot ulcers, - surgical procedures related to PAD - coronary revascularization   Surgical procedures includes: percutaneous transluminal angioplasty, peripheral artery bypass, thrombectomy, thrombolysis, amputations.  Coronary revascularization includes: percutaneous coronary intervention (PCI), coronary artery bypass graft. |
| Units of measure: Measured in count of events. | Time Frame: From baseline to 3 years of FU. |
| To compare the occurrence of macrovascular complications in the lower extremities, in patients receiving optimal diabetes care in a Cardio-Metabolic Clinic versus usual care | - Change in ankle-brachial pressure index (ABI) |
| Units of measure: Measured in ratio to baseline. | Time Frame: From baseline to 3 years of FU. |
| To compare change in protocol-driven medication in patients receiving optimal diabetes care in a Cardio-Metabolic Clinic versus usual care | Change in protocol-driven medication:   - Lipid lowering medication - Antihypertensive medication - Anti-thrombotic medication - Anti-diabetic medication - Nephro-protective medication |
| Units of measure: Measured in percentage [%]. | Time Frame: From baseline to 3 years of FU. |
| To compare change in patient experienced symptoms in patients receiving optimal diabetes care in a Cardio-Metabolic Clinic versus usual care | - Change in symptoms as reported by patients using the Kansas City Cardiomyopathy Questionnaire (KCCQ) |
| Units of measure: Measured in score points (change in percentage [%]). | Time Frame: From baseline to 3 years of FU. |
| To compare the net cost of implementing a Cardio-Metabolic Clinic in patients receiving optimal diabetes care versus usual care | - Net cost of a Cardio-Metabolic Clinic:  Cost of Cardio-Metabolic Clinic minus averted costs   Averted costs includes: averted admissions, medical treatment, and productivity). |
| Units of measure: Measured in dollars [$]. | Time frame: From baseline to 5 + 10 years of FU. |
| To compare the health outcomes of implementing a Cardio-Metabolic Clinic in patients receiving optimal diabetes care versus usual care | - Change in health outcomes measured by quality-adjusted life years (QALY) |
| Units of measure: Measured in score points (change in percentage [%]). | Time frame: From baseline to 5 + 10 years of FU. |
| To compare the cost-effectiveness of implementing a Cardio-Metabolic Clinic in patients receiving optimal diabetes care versus usual care | - Cost-effectiveness ratio of a Cardio-Metabolic Clinic: Net cost/change in health outcomes |
| Units of measure: Measured in $/QALY. | Time frame: From baseline to 5 + 10 years of FU. |

Statistics
**Sample size estimation:**
We anticipate a reduction in the primary endpoint of 15 % in patients with T2D assessed in the Cardio-Metabolic Clinic compared to the standard of care care. With a power of 80% and an alpha value of 0.05.
1306 patients are needed. We anticipate a dropout rate around 10-15% and therefore 1500 patients will have to be included in the study.

**Descriptive statistics**
Flow of participants will be described with a flowchart for group allocation.
Baseline characteristics between the control group/intervention group will be summarized as mean (SD), median (interquartile range) for continuous variables and as counts (percentages) for categorical variables. Differences between the two groups will be compared with a Wilcoxon rank-sum test for continuous variables and Pearsons Chi2/ Fisher's exact test for categorical variables. Missing data will be specified. Study objectives will be analyzed using the intention-to-treat principle.

**Analysis of primary and secondary outcomes**
The primary and secondary outcome will be analyzed using cumulative incidence function for the composite primary outcome as well as each composite secondary outcome.
Furthermore, we will investigate CV cause mortality through Kaplan Meier estimates.

**Analysis of numerical outcome**
For each supportive numerical outcome, we will utilize mixed effects linear regression with included fixed effect for time point and baseline value of the outcome. For each linear mixed effects model, a random intercept will be included for each enrolled patient. These outcomes will be measured at baseline and at 3-year follow-up for the group randomized to drug optimization.
Examples of numerical outcome: changes in biochemistry from baseline to follow-up.

**Analysis of categorical outcome**
For each supportive categorical outcome, multivariable logistic regression with generalized estimating equations (GEE), allowing analysis of repeated measures, will be applied, including adjustment for baseline factors, which may contribute to the outcome variable. The model will deliver estimated adjusted odds ratio and 95% confidence interval.
Examples of supportive categorical outcomes: de novo/worsening retinopathy, nephropathy, hypertension and hypercholesterolemia.

**Testing and confounders**
The statistical test will be performed in a hierarchical order according to the succession of the objectives. The p-value will be corrected with the Bonferroni-Holm method.
Potential effect modifiers are: Sex, age, baseline treatment regime, and baseline comorbidities.

# Ethics and dissemination

**Ethics**

The study complied with the Declaration of Helsinki, and was approved by the local ethics committee (Project-ID: S-20230015). When participating in this study, the intervention will consist of an intensive effort in diabetes care based on the most recent guidelines and recommendations. Whereas, the control group will receive the standard of care from their regular physician(s). Therefore, in theory, the treatment should be the same, and we do not anticipate any associated risks. However, we anticipate that a comprehensive evaluation and examination will lead to significant differences in the treatment regimen. Considering this, we expect a therapeutic gain, both in CVD death and diabetic microvascular complications for the participants receiving the intervention. The participants in the control group will receive the standard of care, thus, there are no ethical considerations.
The local ethical committee will be notified of a protocol amendment if any changes to the research project could potentially affect the participants.

The national patient insurance will cover the participants. No compensation or remuneration will be provided for participation in the study.

**Data protection and access**

The law regarding General Data Protection Regulation and the Data Protection Act will be followed in the management of personal information (i.e. journal or health information). All information will be stored according to the National Danish Data Protection Agency and only persons with secrecy and connection to the study will access the data. Permission to process and store data was granted by the regional data protection agency at Odense University Hospital, and participant data will be stored using this electronic case-report form in REDCap.

**Dissemination policy**

We will make efforts to publish all the results from this study in international peer-reviewed journals. Study information will be disclosed at ClinicalTrials.gov, and regardless of whether the outcomes are positive, negative, or inconclusive, they will be published on the website (www.clinicaltrial.gov). Participant data will be analyzed as groups, ensuring their confidentiality and preventing identification by others. Authorship of publications will be in accordance with the Vancouver style by the International Committee of Medical Journal Editors.

Financing The Cardiovascular Research Unit has taken the initiative for this research project.
External funding of 1.260.000 kr was received from ”Ramme for højt specialiseret funktion og strategiske indsatsområder” from OUH/Region South Denmark.
 Applications for further financing will be applied.

# References

1. Copenhagen SDC. Diabetes i tal - Videncenter for Diabetes [updated 10. december 2020. Available from: <https://videncenterfordiabetes.dk/viden-om-diabetes/generelt-om-diabetes/diabetes-i-tal#videnskaben-bag-63>.

2. Mellbin LG, Anselmino M, Lars R. Diabetes, prediabetes and cardiovascular risk. European Journal of Cardiovascular Prevention &amp; Rehabilitation. 2010;17(1_suppl):s9-s14.

3. Arnold SV, Kosiborod M, Wang J, Fenici P, Gannedahl G, LoCasale RJ. Burden of cardio-renal-metabolic conditions in adults with type 2 diabetes within the Diabetes Collaborative Registry. Diabetes Obes Metab. 2018;20(8):2000-3.

4. Dunlay SM, Givertz MM, Aguilar D, Allen LA, Chan M, Desai AS, et al. Type 2 Diabetes Mellitus and Heart Failure: A Scientific Statement From the American Heart Association and the Heart Failure Society of America: This statement does not represent an update of the 2017 ACC/AHA/HFSA heart failure guideline update. Circulation. 2019;140(7).

5. Gæde P, Vedel P, Larsen N, Jensen GVH, Parving H-H, Pedersen O. Multifactorial Intervention and Cardiovascular Disease in Patients with Type 2 Diabetes. New England Journal of Medicine. 2003;348(5):383-93.

6. Davies MJ, D’Alessio DA, Fradkin J, Kernan WN, Mathieu C, Mingrone G, et al. Management of Hyperglycemia in Type 2 Diabetes, 2018. A Consensus Report by the American Diabetes Association (ADA) and the European Association for the Study of Diabetes (EASD). Diabetes Care. 2018;41(12):2669-701.

7. Mann JF, Schmieder RE, McQueen M, Dyal L, Schumacher H, Pogue J, et al. Renal outcomes with telmisartan, ramipril, or both, in people at high vascular risk (the ONTARGET study): a multicentre, randomised, double-blind, controlled trial. Lancet. 2008;372(9638):547-53.

8. de Vries FM, Kolthof J, Postma MJ, Denig P, Hak E. Efficacy of standard and intensive statin treatment for the secondary prevention of cardiovascular and cerebrovascular events in diabetes patients: a meta-analysis. PLoS One. 2014;9(11):e111247.

9. Fung CSC, Chin WY, Dai DSK, Kwok RLP, Tsui ELH, Wan YF, et al. Evaluation of the quality of care of a multi-disciplinary risk factor assessment and management programme (RAMP) for diabetic patients. BMC Family Practice. 2012;13(1):116.

10. Wan EYF, Fung CSC, Jiao FF, Yu EYT, Chin WY, Fong DYT, et al. Five-Year Effectiveness of the Multidisciplinary Risk Assessment and Management Programme–Diabetes Mellitus (RAMP-DM) on Diabetes-Related Complications and Health Service Uses—A Population-Based and Propensity-Matched Cohort Study. Diabetes Care. 2017;41(1):49-59.

11. Zinman B, Wanner C, Lachin JM, Fitchett D, Bluhmki E, Hantel S, et al. Empagliflozin, Cardiovascular Outcomes, and Mortality in Type 2 Diabetes. New England Journal of Medicine. 2015;373(22):2117-28.

12. Neal B, Perkovic V, Matthews DR. Canagliflozin and Cardiovascular and Renal Events in Type 2 Diabetes. N Engl J Med. 2017;377(21):2099.

13. Wiviott SD, Raz I, Bonaca MP, Mosenzon O, Kato ET, Cahn A, et al. Dapagliflozin and Cardiovascular Outcomes in Type 2 Diabetes. New England Journal of Medicine. 2019;380(4):347-57.

14. McMurray JJV, Solomon SD, Inzucchi SE, Køber L, Kosiborod MN, Martinez FA, et al. Dapagliflozin in Patients with Heart Failure and Reduced Ejection Fraction. New England Journal of Medicine. 2019;381(21):1995-2008.

15. Packer M, Anker SD, Butler J, Filippatos G, Pocock SJ, Carson P, et al. Cardiovascular and Renal Outcomes with Empagliflozin in Heart Failure. New England Journal of Medicine. 2020;383(15):1413-24.

16. Marso SP, Daniels GH, Brown-Frandsen K, Kristensen P, Mann JFE, Nauck MA, et al. Liraglutide and Cardiovascular Outcomes in Type 2 Diabetes. New England Journal of Medicine. 2016;375(4):311-22.

17. Marso SP, Bain SC, Consoli A, Eliaschewitz FG, Jódar E, Leiter LA, et al. Semaglutide and Cardiovascular Outcomes in Patients with Type 2 Diabetes. New England Journal of Medicine. 2016;375(19):1834-44.

18. Gerstein HC, Colhoun HM, Dagenais GR, Diaz R, Lakshmanan M, Pais P, et al. Dulaglutide and cardiovascular outcomes in type 2 diabetes (REWIND): a double-blind, randomised placebo-controlled trial. Lancet. 2019;394(10193):121-30.

19. A/S NN. Semaglutide 2.4 mg reduces the risk of major adverse cardiovascular events by 20% in adults with overweight or obesity in the SELECT trial Company Announcement2023.

20. Gyberg V, De Bacquer D, De Backer G, Jennings C, Kotseva K, Mellbin L, et al. Patients with coronary artery disease and diabetes need improved management: a report from the EUROASPIRE IV survey: a registry from the EuroObservational Research Programme of the European Society of Cardiology. Cardiovasc Diabetol. 2015;14:133.

21. Reiter-Brennan C, Dzaye O, Davis D, Blaha M, Eckel RH. Comprehensive Care Models for Cardiometabolic Disease. Curr Cardiol Rep. 2021;23(3):22.

22. Pagidipati NJ, Nelson AJ, Kaltenbach LA, Leyva M, McGuire DK, Pop-Busui R, et al. Coordinated Care to Optimize Cardiovascular Preventive Therapies in Type 2 Diabetes: A Randomized Clinical Trial. JAMA. 2023;329(15):1261-70.

23. Manla Y, Almahmeed W. Cardiometabolic Clinics: Is There a Need for a Multidisciplinary Clinic? Frontiers in Clinical Diabetes and Healthcare. 2022;3.

24. Stol DM, Over EAB, Badenbroek IF, Hollander M, Nielen MMJ, Kraaijenhagen RA, et al. Cost-effectiveness of a stepwise cardiometabolic disease prevention program: results of a randomized controlled trial in primary care. BMC Med. 2021;19(1):57.

25. American Diabetes A. 2. Classification and Diagnosis of Diabetes: Standards of Medical Care in Diabetes-2021. Diabetes Care. 2021;44(Suppl 1):S15-S33.

26. Collet JP, Thiele H, Barbato E, Barthélémy O, Bauersachs J, Bhatt DL, et al. 2020 ESC Guidelines for the management of acute coronary syndromes in patients presenting without persistent ST-segment elevation. Eur Heart J. 2021;42(14):1289-367.

27. Knuuti J, Wijns W, Saraste A, Capodanno D, Barbato E, Funck-Brentano C, et al. 2019 ESC Guidelines for the diagnosis and management of chronic coronary syndromes: The Task Force for the diagnosis and management of chronic coronary syndromes of the European Society of Cardiology (ESC). European Heart Journal. 2019;41(3):407-77.

28. Adams HP, Jr., Bendixen BH, Kappelle LJ, Biller J, Love BB, Gordon DL, et al. Classification of subtype of acute ischemic stroke. Definitions for use in a multicenter clinical trial. TOAST. Trial of Org 10172 in Acute Stroke Treatment. Stroke. 1993;24(1):35-41.

29. Gerhard-Herman MD, Gornik HL, Barrett C, Barshes NR, Corriere MA, Drachman DE, et al. 2016 AHA/ACC Guideline on the Management of Patients With Lower Extremity Peripheral Artery Disease: A Report of the American College of Cardiology/American Heart Association Task Force on Clinical Practice Guidelines. Circulation. 2017;135(12):e726-e79.

30. McDonagh TA, Metra M, Adamo M, Gardner RS, Baumbach A, Böhm M, et al. 2021 ESC Guidelines for the diagnosis and treatment of acute and chronic heart failure. Eur Heart J. 2021;42(36):3599-726.

31. Hindricks G, Potpara T, Dagres N, Arbelo E, Bax JJ, Blomström-Lundqvist C, et al. 2020 ESC Guidelines for the diagnosis and management of atrial fibrillation developed in collaboration with the European Association for Cardio-Thoracic Surgery (EACTS): The Task Force for the diagnosis and management of atrial fibrillation of the European Society of Cardiology (ESC) Developed with the special contribution of the European Heart Rhythm Association (EHRA) of the ESC. Eur Heart J. 2021;42(5):373-498.

32. Marx N, Federici M, Schütt K, Müller-Wieland D, Ajjan RA, Antunes MJ, et al. 2023 ESC Guidelines for the management of cardiovascular disease in patients with diabetes: Developed by the task force on the management of cardiovascular disease in patients with diabetes of the European Society of Cardiology (ESC). European Heart Journal. 2023;44(39):4043-140.

33. Bojer AS, Hansen K B, Egstrup K, Jensen M T , Krarup N T, Snorgaard O, Rossing P, Knudsen S P. 26. Diabetes og hjertesygdom: Danish Cardiologic Society 2022 [Available from: <https://nbv.cardio.dk/diabetes>.

34. Hansen K B KJK, Balasubramaniam K, Bjerregaard-Andersen M, Breum L. Type 2 Diabetes: Danish Endocrine Society (DES); 2011 [updated Oct 2022.

35. Sundhedsstyrelsen. Sundhedsstyrelsens udmeldinger om indtag af alkohol. In: Sundhedsstyrelsen, editor. 2022.

36. Dalgaard F. RN, Kristiansen S. B., Darkner S. 15. Atrieflimren og atrieflagren Cardio.dk: Danish Cardiologic Society; 2022 [Available from: <https://nbv.cardio.dk/af>.

37. Sandholt B V GN, Eldrup N, Jørgensen T M M. 33. Perifer arteriesygdom: Danish Cardiologic Society 2022 [Available from: <https://nbv.cardio.dk/pad>.

38. Sundhedsstyrelsen. Personer med kronisk sygdom tilbydes vaccination mod influenza 2022 [Available from: <https://www.sst.dk/da/Influenza/Personer-med-kronisk-sygdom>.

39. Sundhedsstyrelsen. Skal jeg vaccineres? In: Sundhedsstyrelsen, editor. 2022.
